# The Biology of Varicella-Zoster Virus Replication in the Skin

**DOI:** 10.3390/v14050982

**Published:** 2022-05-06

**Authors:** Cristina Tommasi, Judith Breuer

**Affiliations:** 1 School of Cellular and Molecular Medicine, University of Bristol, Bristol BS8 1TD, UK; 2 Department of Infection, Institute of Child Health, University College London, London WC1N 1EH, UK

**Keywords:** varicella-zoster virus, skin, epidermis, keratinocytes, epidermal differentiation, cutaneous blistering lesions, keratins, autophagy, immunity, vaccine

## Abstract

The replication of varicella-zoster virus (VZV) in skin is critical to its pathogenesis and spread. Primary infection causes chickenpox, which is characterised by centrally distributed skin blistering lesions that are rich in infectious virus. Cell-free virus in the cutaneous blistering lesions not only spreads to cause further cases, but infects sensory nerve endings, leading to the establishment of lifelong latency in sensory and autonomic ganglia. The reactivation of virus to cause herpes zoster is again characterised by localised painful skin blistering rash containing infectious virus. The development of *in vitro* and *in vivo* models of VZV skin replication has revealed aspects of VZV replication and pathogenesis in this important target organ and improved our understanding of the vaccine strain vOKa attenuation. In this review, we outline the current knowledge on VZV interaction with host signalling pathways, the viral association with proteins associated with epidermal terminal differentiation, and how these interconnect with the VZV life cycle to facilitate viral replication and shedding.

## 1. Introduction

Varicella-zoster virus (VZV) is a member of the human alphaherpesvirus family. Primary VZV infection causes chickenpox (varicella) in susceptible individuals, which typically manifests in immunocompetent individuals as a skin blistering rash preceded by flu-like symptoms and is usually self-limiting. In immunocompromised individuals, pregnant women as well as healthy adults, VZV infection may cause serious illness and even be lethal, often as a consequence of bacterial superinfections. A vaccine for VZV exists, which is based on the live attenuated parental VZV Oka strain [1,2]. Because the vaccine virus is live, it is not suitable for individuals with compromised immune systems. In addition, it can sometimes cause rashes, establishes latency in sensory nerves and reactivates in a small number of cases to cause herpes zoster (HZ). Consequently, research for alternative VZV vaccines, and therefore for a better understanding of VZV life cycle, is warranted.

VZV is transmitted between hosts by contact with the cutaneous blistering lesions containing the highly infectious cell-free virus or through inhalation of the aerosolized virions released from the skin lesions and to some extent the respiratory tract. During primary infection, VZV initially infects cells of the mucosa lining the upper respiratory tract where it is initially detected by dendritic cells (DCs) and transported to lymphoid tissues, primarily the tonsils, where it infects skin homing markers expressing T-lymphocytes that then deliver it to the skin [3,4]. The virus replicates in the skin epidermis before being released as cell-free virus from the blistering lesions. While replicating in the epidermis, the virus can gain access to the terminal endings of local sensory nerves and travel retrogradely through the axon to the cell bodies where it establishes latency. When reactivated, VZV travels within the axon in anterograde manner to reach the innervated dermatome where it causes herpes zoster (HZ) or shingles, characterised by a localised painful vesicular rash [4,5].

The tropism of VZV for the skin is well recognised, however our understanding of the molecular mechanisms of VZV replication in the epidermal tissue of the skin are still incomplete. VZV being a human-restricted virus with limited infectivity for other species, has limited the use of animal models for the study of viral natural history and pathogenicity. Most research into VZV cellular interactions has been in human embryonic lung fibroblasts (HELF), human lung MRC-5 fibroblasts and the human melanoma MeWo cell line. While these cell types are relatively easy to infect with VZV, none of them is representative of the human epidermis, which is the actual site of much VZV replication and shedding. An *in vivo* VZV infection model that closely mimics VZV infection of human skin *in vivo* was developed by the Arvin group and is based on the infection with VZV of xenografts of human fetal skin implanted into SCID-hu mice [3,6,7]. VZV infection of hTERT and human primary keratinocytes, organotypic human skin culture and human skin explants, also provides better *in vitro* representation of VZV infection of the epidermis [8,9,10,11,12,13]. The calcium-switch model of VZV infection, which allows analysis of VZV during keratinocyte differentiation provides a useful 2D *in vitro* model of VZV infection in human epidermis [8,9].

This review will explore what is understood to date about the mechanisms of VZV replication in the epidermis of the skin, as discovered in the very few existing human skin models of VZV infection.

## 2. VZV Life Cycle

During primary infection, VZV infects epithelial cells of the upper respiratory tract mucosa. It is believed that at this location the virus encounters and infects DCs, which then travel to the tonsils and other regional lymphoid tissues, where they transfer the virus to T lymphocytes [14,15]. It is not clear how T cells get infected by DCs, however it is well recognised that T lymphocytes play a crucial role in the dissemination of the virus from the initial sites of infection to the rest of the body, particularly the skin, during the viraemic stage of infection. VZV-infected T cells are mainly memory CD4^+^ T cells expressing activation and skin-homing markers, such as CC-chemokine receptor 4 (CCR4) and cutaneous leukocyte antigen (CLA), but VZV also infects and activates naïve T cells [3,16]. It has been demonstrated *in vitro* that natural killer (NK) cells can also be infected by VZV and consequently increase the expression of skin-homing proteins [17]. However, their role in VZV skin pathogenesis *in vivo* and whether they contribute to the virus dissemination to T cells has not yet been elucidated. The incubation period during VZV infection is about 10–21 days, which is the time between VZV infection and the manifestation of symptoms, including the skin vesicular rash. Studies in SCID-hu mice models indicate that T cells reach the skin within days after infection, but it appears that the virus is able to evade initial antiviral responses through its interaction with interferon (IFN)-mediated innate immunity and apoptotic pathways (reviewed in [4]). These cause the virus to remain undetected for several days and therefore have enough time to replicate and produce the infectious virions-filled cutaneous blistering lesions that are needed for spread to new hosts [18,19]. During replication in the skin, VZV infects the terminal endings of sensory ganglia, which innervate the skin dermis and epidermis. It is believed that the intraepidermal nerve fibres encounter VZV at the level of the cutaneous lesions and are thereby infected by the cell-free virus [20,21]. Infection of neurons by cell-free virus allows the establishment of latency, whereas infection with cell-associated virus results in lytic infection [20,22,23]. VZV then travels to the neuronal cell bodies through retrograde axonal transport and there remains latent [24,25]. An additional possible method of neurons infection may involve the direct transmission of the virus from VZV-infected T cells [16] to neuronal cell bodies, but the precise mechanisms for this are still unclear [26]. Once reactivated, VZV travels back to the skin causing HZ, which is characterised by a painful blistering rash confined to the skin area innervated by the ganglion where reactivation occurred [5].

## 3. VZV and Skin

### 3.1. The Skin

The skin represents the outermost organ of the human body and encompasses the following tissues from the innermost to the outermost: hypodermis, dermis and epidermis. The dermis and epidermis represent the skin connective and epithelial tissues, respectively. While VZV replicates both the in the dermis and the epidermis, the latter represents the major site of viral replication, as well as the location where blistering lesions laden with infectious VZV virions form, guaranteeing virus transmission to new susceptible hosts.

**Figure 1:**
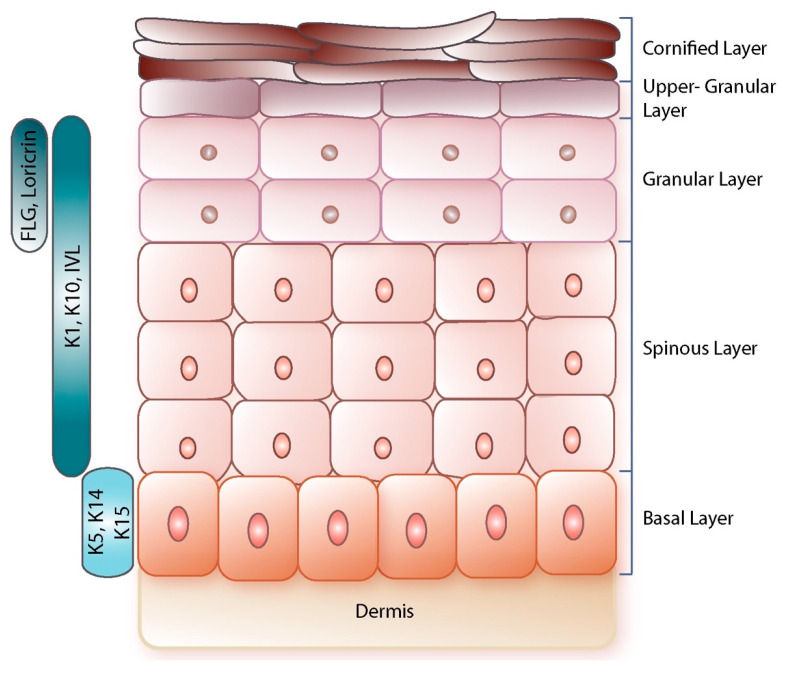
Schematic of the human epidermis. The human epidermis is a pluristratified tissue made up of basal, spinous, granular, upper-granular and cornified layers, in ascending order. The keratinocytes in the basal layer express keratins K5 and K14, as well as K15, which are then substituted by markers of epidermal differentiation such as K1, K10, IVL in the spinous layer. With progression of differentiation, other markers such as FLG and loricrin are expressed in the granular layer. The keratinocytes at the level of the cornified layer are called corneocytes as they are devoid of organelles and nuclei and are finally sloughed off.

The keratinocytes, which are the main cell type in the epidermis, are subject to cycles of proliferation, differentiation and death that allow preservation of epidermal homeostasis as well as of epidermal barrier function [27,28,29]. They maintain their proliferative capacity in the basal epidermal layer and when starting to differentiate they cease dividing and move to occupy the suprabasal spinous, granular, upper-granular and cornified layers (Figure 1). In the suprabasal layers, the keratinocytes express markers of terminal differentiation, until they ultimately loose nuclei and organelles and become corneocytes. Corneocytes are essentially bundles of filaments surrounded by cross-linked proteins and insoluble lipids and are ultimately shed out as dead cells [27,29,30]. Among the proteins that keratinocytes express during the distinct stages of differentiation, there are keratins, which are usually organised in heterodimers to constitute the keratin intermediate filaments (KIFs) that extend from the cell nucleus to the cell-cell desmosomes junctions. The basal keratinocytes typically express keratin 5 (K5) and 14 (K14). With differentiation, K5 and 14 are replaced by K1 and K10 and other “specialised” keratins such as K9 in the palms and soles’ skin and K2 in thickened skin areas [31,32]. Keratin 15 (K15), which in the hair follicles (HFs) is associated with bulge stem cells, is also expressed by undifferentiated keratinocytes and lost when keratinocytes differentiate [32]. Other proteins that mark epidermal terminal differentiation include, but are not limited to, involucrin (IVL), loricrin, filaggrin (FLG), trichohyalin (TCHH) and small proline-rich proteins (SPRRs) [27].

### 3.2. VZV Skin Tropism

The earliest histological evidence of VZV infection from skin biopsies of both chickenpox and HZ is observed in the HFs [33,34]. This was also detected in skin xenografts on SCID-hu mice, where 24 h post systemic injection of VZV-infected T cells, a high number of T cells was observed in the HFs’ skin cells, as well as in basal epidermal keratinocytes [18]. It is likely that VZV-carrying T lymphocytes reach the skin cells surrounding the HFs bulb through the extensive microvasculature which wraps around, possibly by diapedesis, however this has not been formally demonstrated [4]. The mechanisms whereby the virus is then transferred from T cells to HFs’ skin cells are not known. Based on mathematical modelling applied to cutaneous blistering lesions following VZV vaccination, it has been inferred that each skin lesion arises from no more than three virions [35]. It could be hypothesised therefore that one virion (three at most) is transferred from a T cell to a skin cell and from there replicates to generate one blister. It is also unclear which are the exact skin epithelial cells targeted by the virus at the level of the HFs and how the virus is then transferred from the HFs to the interfollicular basal keratinocytes.

The virus replicates, spreads among cells and is highly cell-associated in the interfollicular basal keratinocytes, but becomes infectious only when keratinocytes differentiate into the upper epidermal layers, where it accumulates as infectious cell-free virions in the cutaneous blistering lesions [4,8,20,36]. Evidence from VZV infection of human keratinocytes in the calcium-switch model shows an increase in viral copy number occurring with epidermal differentiation, and this in turn is associated with increased expression of VZV genes classed as early or late in the replication cycle, with the loss of immediate early gene expression. Specifically, VZV immediate early genes are generally expressed in the basal epidermal layer, whereas late genes, such as the glycoproteins gC and gE, tend to be expressed in the suprabasal epidermal layers [8]. This provides some indication that VZV depends upon epidermal terminal differentiation to fully mature into cell-free and infectious virions that can be released by the skin lesions for infection of new hosts. The “physical” barrier represented by epidermal terminal differentiation of keratinocytes, as differentiated keratinocytes are more resistant to infection, may contribute to blisters remaining discrete lesions in the skin. However, a major contribution to this state is likely to come from the induction of IFNα and IFNβ, as well as immunity regulators, such as phosphorylated signal transducer and activator of transcription 1 (pSTAT1) and NF-κB in bystander epidermal cells, that also contain viral spread in the skin [4,18].

From studies conducted mainly in MeWo cells and lung fibroblasts, it has been determined that VZV enters the cell through initial interaction with cell surface heparan sulfate proteoglycans (HSPGs) [37], followed by binding to additional receptors, namely the insulin-degrading enzyme (IDE) and the mannose 6-phosphate receptor (MPR) [37,38]. The binding to these receptors is mediated by a number of viral glycoproteins. Specifically, as the MPR interacts with mannose-6-phopsphate groups, it can be bound by the glycoproteins gB, gI, gH, gE [36,39], whereas the IDE receptor can be bound by gE [38,40]. However, the role of gE-IDE in mediating cell-to-cell spread is still debated as it has been reported by later studies that IDE binds a precursor rather than a mature form of the gE protein and that this interaction occurs in the cell cytoplasm [41]. The roles of these receptors for VZV ingress into keratinocytes and for viral cell-to-cell spread in the epidermis are still unclear. It has been shown that the HSPGs and the MPR receptors are expressed in the basal epidermal layer and lost in the suprabasal layers [36,42]. However, viral glycoproteins are usually expressed only when infected keratinocytes differentiate [8], which would limit their interaction with MPR receptors at the level of the basal undifferentiated keratinocytes. Hence, further investigation in skin models of VZV infection will be necessary to determine whether these are the sole receptors needed by VZV for infection and to evaluate how their differential expression throughout the diverse epidermal layers is used by VZV in its life cycle. MPRs have also been implicated in the generation of cell-free VZV virions in the epidermal suprabasal layers, because MPRs also line the vesicles that transport newly enveloped virions to the plasma membrane and late endosomes [36,37,43]. In this study, the transport of virions to late endosomes was interpreted to result in virion degradation in the basal epidermal layer. In contrast, the absence of MPRs in the upper epidermal layers would prevent their transit to late endosomes, therefore allowing their maturation into cell-free infectious virions [36]. However, further studies are required to understand how cell-free VZV virions are generated in the epidermis and to understand whether autophagy (which is discussed in paragraph 4.2) may play a role in this process.

### 3.3. Viral Components of VZV Replication in the Skin

Early work using skin grafted into SCID-hu mice [3,6,7] showed differences between the behaviour of VZV in cells monolayers, even in the case of skin-derived cells, and differentiated epidermis. Using deletion mutagenesis in SCID-hu mice skin xenografts, a number of VZV proteins which appear to be dispensable for VZV infection in cell monolayers, were identified as essential to VZV pathogenesis *in vivo* (reviewed in [4]). Examples include the viral glycoprotein gC (encoded by ORF14), gM (encoded by ORF50), the regulatory protein ORF10 and the kinase ORF47, [6,7,44,45,46]. Further viral proteins that are essential to VZV skin pathogenesis *in vivo* encompass proteins encoded by the ORF9–12 gene cluster and the transcriptional transactivators IE62 (encoded by ORF62 and ORF71) and IE63 (encoded by ORF63 and ORF70), although for IE63 the relevance in VZV infection *in vivo* is dependent on the phosphorylation status [4,45,47]. The glycoproteins that are essential in skin pathogenesis *in vivo* encompass the glycoprotein gE (encoded by ORF68), which is essential for VZV replication and cell–cell spread in skin [48,49], and its partner gI [50], as well as glycoproteins gB (encoded by ORF31) and gH (encoded by ORF 37), which, in a complex with gL (encoded by ORF60), regulate both viral ingress into the cell and cell–cell fusion to generate syncytia [51,52,53,54,55]. Syncytia production is a hallmark of VZV infection and it is caused by the fusion of infected with uninfected cells to form polykaryocytes [4,52,53]. The fusion process is triggered by the interaction of gB and gH/gL, which for this purpose localise at the surface of infected cells, with receptors on the cell membranes of neighbouring cells. First, a fusion pore between adjacent cells membranes is formed, then the membranes fully fuse and combine their cytoplasm and nuclei to generate the multinucleated structure typical of syncytia (reviewed in [56]). The cytoplasmic domain of gB is indispensable for the fusion process, particularly the immunoreceptor tyrosine- based inhibition motif (ITIM) [52] and the lysine cluster downstream of the ITIM [57]. It is important to note that syncytia formation relies on a fine balance of cell-cell fusion; too little fusion would not allow sufficient cell-to-cell spread, whereas too much fusion would be detrimental for the virions’ assembly and replication. The process of fusion therefore needs to be tightly controlled by the virus, probably through manipulation of host proteins, such as the phosphatase calcineurin [56].

## 4. VZV Interaction with Host Epidermal Pathways

### 4.1. VZV Interplay with Epidermal Terminal Differentiation

**Figure 2:**
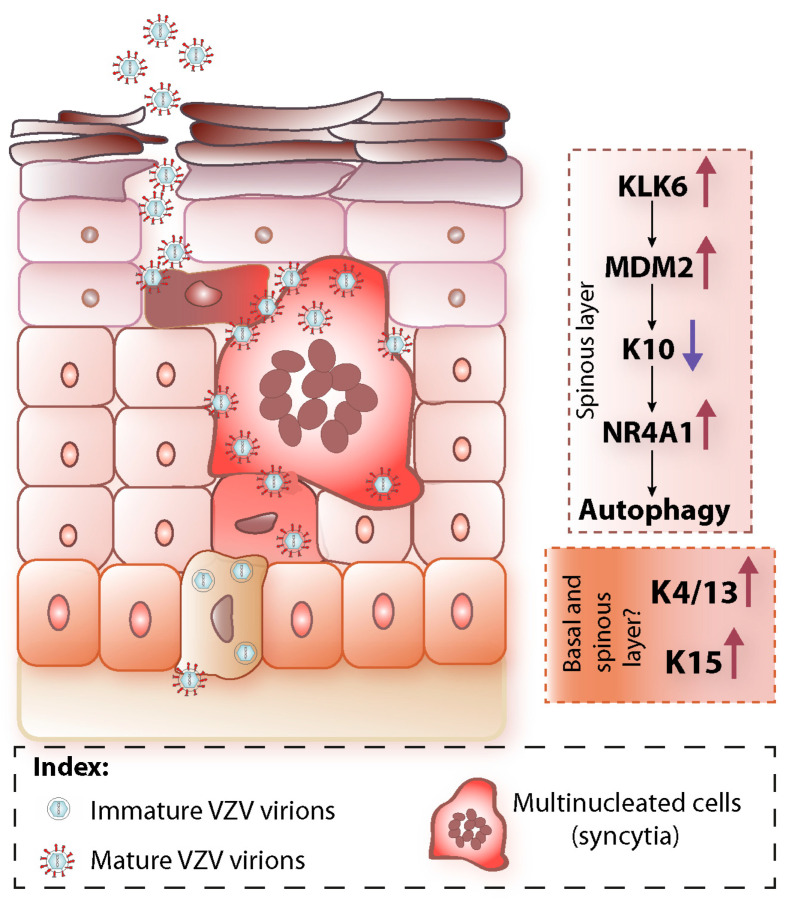
Schematic of VZV interplay with epidermal terminal differentiation. Left model of VZV-infected epidermis: VZV infects epidermal basal keratinocytes where starts to replicate. The newly formed virions in the basal keratinocytes are immature and do not express glycoproteins. In the suprabasal layers VZV virions mature together with epidermal differentiation and express glycoproteins. They also spread cell-to-cell via syncytia formation. At the uppermost epidermal layers mature and cell-free virions are released. Right panel: summary of the major changes induced by VZV to epidermal host pathways. They include in the spinous layer the downregulation of keratin K10 due to its degradation and caused by MDM2, which is upregulated by increased levels of KLK6. K10 downregulation has a structural effect on blister and syncytia formation, but also a signalling effect by upregulating NR4A1, which in turn induces activation of autophagy pathways. Keratin K15, which is normally expressed in the basal layer, is upregulated and its expression is found also in differentiated keratinocytes. K4 and K13 are also upregulated in differentiated keratinocytes during VZV infection.

Like other skin epitheliotropic viruses, e.g., cutaneous human papilloma viruses (HPVs) [58,59], VZV life cycle and epidermal terminal differentiation are tightly interconnected. VZV profoundly alters gene and protein expression in the host keratinocytes, particularly in pathways that are unique to the epidermis, namely epidermal differentiation and epidermal barrier function pathways [8,9] (Figure 2).

In *in vitro* models of VZV infection of human epidermis, it was demonstrated that VZV induces the degradation of the suprabasal epidermal differentiation marker K10, as well as the downregulation of a number of corneodesmosomal proteins [8,9]. As K10, together with K1, forms the core of the intermediate filament network of the suprabasal keratinocytes and is a crucial component of the epidermal barrier function [60,61,62], its degradation is probably caused by VZV to facilitate blistering lesion formation. Indeed, the disruption of keratins is often associated with skin blistering conditions [63].

VZV keratinocytes infection is also characterised by upregulation of a number of proteases, most notably kallikreins [8]. Kallikreins are serine proteases, whose most recognised role in skin is mediating epidermal desquamation through the degradation of corneodesmosomal proteins [64,65]. Kallikrein 5 (KLK5) and 7, whose role in epidermal desquamation is very well established, are highly upregulated in VZV-infected and differentiated keratinocytes, concomitant with downregulation of some of their known targets, notably desmoglein-1 (DSG1), desmocollin-1 (DSC1) [8,66]. It can be hypothesised that, through the upregulation of KLK5 and KLK7 levels, VZV exploits the process of epidermal desquamation for its shedding from the cornified envelope. Notably, it was observed that although KLK5 and KLK7 are highly upregulated in VZV keratinocytes infection, the most upregulated kallikreins are KLK6, 12 and 13, which are not strictly associated with skin desquamation [8]. In fact, an important role for KLK6 in VZV epidermal infection appears to be to upregulate cytoplasmic levels of the murine double minute 2 (MDM2) protein, allowing MDM2 to bind and ubiquitinate K10 and target it for proteosome degradation [9].

The formation of syncytia structures is well documented in MeWo cells [56], and multinucleated cells are also observed in VZV-infected keratinocytes, which have been differentiated with calcium-switch [9], as well as in skin from the SCID-hu mouse model [52]. However, the molecular mechanisms by which VZV overcomes host keratinocyte barriers to induce fusion have not yet been elucidated and warrant further investigation. We observed that in infected keratinocytes MDM2 interacts with K10 in nascent syncytial structures. The interaction is absent in larger syncytia, by which stage K10 is already lost, suggesting that K10 degradation may also contribute to syncytia expansion [9]. Indeed, the disruption of KIFs would lead to a disruption of cell–cell junctions, which may facilitate cell–cell fusion. Consistent with this, VZV infection of keratinocytes is characterised by an extensive downregulation of adhesion proteins [8].

K10 not only serves a structural function in VZV infection of the epidermis. In line with increasing studies reporting involvement of keratins in gene expression and signalling pathways [67,68], K10 degradation was found to also have a signalling role in VZV infection of epidermis, by upregulating the expression of the transcription factor nuclear receptor subfamily 4 group A member 1 (NR4A1) [9,69]. NR4A1 expression is non-nuclear in VZV-infected keratinocytes [9], which has been associated with a non-transcriptional role of NR4A1, for example in autophagy pathways [70]. In the case of VZV skin infection, cytoplasmic NR4A1 induces autophagy activation [9].

In the transcriptional signature of VZV-infected keratinocytes, *KRT4/13* (which are mucosal keratins, [32]) and *KRT15* genes are also upregulated [8]. Recent work showing the upregulation of K17 and WNT5a expression [71], together with K15 [8], seems to suggest an involvement of wound healing pathways in VZV infection, but the exact mechanisms and which precise characters are involved are unknown.

### 4.2. VZV Interplay with Epidermal Autophagy

Macroautophagy, hereafter called “autophagy”, is a process involving the degradation and recycling of proteins and cellular components, which is activated in response to cellular stress, e.g., following microbes’ infection, to guarantee cell homeostasis. The autophagy events are mediated by a number of proteins called autophagy-related genes (ATGs), as well as not ATG proteins [72] and involve the formation of a autophagophore which surrounds the to-be-degraded cargo, then matures into a double-membrane autophagosome, which eventually fuses with the lysosome to generate the autolysosome, the place where degradation of the cargo occurs (reviewed in [73,74]). In some cases, autophagosomes may fuse with late endosomes to form amphisomes, before fusion with lysosomes [73,75]. The termination of the different stages of autophagosome maturation, including autophagosome/amphisome fusion with the lysosome, is known as autophagic flux. Autophagy is constitutively operative in the epidermal granular layer [76], where it has a pivotal role in epidermal homeostasis by contributing to epidermal terminal differentiation and barrier function, as also proven by a number of inflammation and skin barrier diseases characterised by defects in autophagy ([77] and reviewed in [78]). Akinduro et al. [76] showed that a type of autophagic nuclear degradation called nucleophagy occurs in keratinocytes at the level of the granular layer, demonstrating that nucleophagy plays a role in the process of nuclei loss during late terminal differentiation.

In VZV-infected and differentiated keratinocytes and skin explants, NR4A1 has been found to be crucial in the induction of autophagy mechanisms, as demonstrated by the decreased expression of key autophagy markers, such as LC3, when NR4A1 was knocked down [9]. Notably, LC3, which in infected cells is widely expressed in the cytoplasm, following NR4A1 downregulation, gets re-localised around the cell nuclei [9], which is a characteristic feature of LC3 in terminally differentiated keratinocytes [76].

The analyses of VZV-infected fibroblasts and MeWo cells, as well as keratinocytes and skin explants, indicate that autophagy has a proviral effect during VZV skin infection and suggest that the virus exploits autophagy events for completion of its life cycle [9,79,80]. It was shown, mostly in MRC-5 fibroblasts and MeWo cells, that, unlike HSV-1, in VZV infection the autophagic flux is not interrupted [81,82]. In fact, no genes with autophagy inhibition function have been so far identified in the VZV genome, whereas the HSV-1 genome expresses at least two autophagy inhibitors genes, ICP34.5 and US11 [83,84]. Notwithstanding, a recent investigation showed in VZV-infected MRC-5 fibroblasts that the late stages of the autophagy flux are inhibited [85]. In keeping with this, in VZV-infected and differentiated keratinocytes lysosomal proteins, such as lysosomal associated membrane protein 2 (LAMP2), and other autolysosome trafficking proteins, such as vesicle-associated membrane protein 7 (VAMP7), are downregulated [9]. It has also been observed, in fibroblasts and MeWo cells, that VZV virions localize in single membranes vesicles, presumably amphisomes, as characterised by both the autophagosome protein LC3 and the endosomal protein Rab11 [86]. Given that the synthesis and maturation of some VZV glycoproteins, such as gE, are reduced when autophagy is inhibited [80], it could be hypothesised that the autophagy machinery is involved in the secondary envelopment of VZV virions or, as hypothesised by Grose et al. [86], in the virions exocytosis pathway, through employment of amphisomes’ vesicles. More future studies will be needed to clarify the molecular mechanisms of autophagy in VZV infection of the skin, to understand whether the virus co-opts amphisomes structures for the completion of its life cycle, also with respect to the specifics of the diverse epidermal layers that the virus navigates through. Many aspects are still unclear and in some cases contradictory. For example, Chen et al. [36] showed that MPR expression is reduced in the suprabasal epidermal layers, yet amphisomes express MPR proteins as they are the result of the fusion of autophagosomes and late endosomes [75].

### 4.3. VZV Interplay with Epidermal Innate Immunity

The major innate immunity cells involved in the reaction to VZV infection, including DCs, Langerhans and NK cells, have been recently reviewed in [87]. Here, we focus on discussing how VZV restrains the innate immune responses that involve cytokines and antiviral proteins produced by keratinocytes upon infection. In studies of VZV-infected skin in the SCID-hu mouse model, it was shown that VZV averts the degradation of inhibitor of NF-κBα (IκBα) and NF-κB is segregated in the cytoplasm of infected cells [88]. Concomitantly, infected keratinocytes also display activation of STAT3 (and therefore increase levels of pSTAT3) which in turn up-regulates the expression of survivin, which blocks host cells’ apoptosis, thereby guaranteeing the virus survival in the host cell [89]. In addition, in the infected keratinocytes, VZV is able to block, through ORF61, the antiviral cellular mechanism induced by IFNs and represented by the protein promyelocytic leukemia protein- nuclear bodies (PML-NB) formation, which entraps nascent virions in the nucleus, thereby preventing them from leaving the nucleus to complete their life cycle [90]. Finally, in fibroblasts and melanoma cells, it was demonstrated that VZV IE62 mediates the block of IFN regulatory factor 3 (IRF3) phosphorylation to prevent IFNβ production [91] and VZV IE63 blocks eukaryotic initiation factor 2 α (eIF-2α) phosphorylation, which is normally a consequence of IFNα activation to inhibit protein translation [92].

## 5. VZV Vaccine and Skin

The varicella vaccine was generated by Takahashi and colleagues in 1974 [2] by serial passaging, first in human embryonic fibroblasts and then in in guinea pig embryo fibroblasts, of VZV virus isolated from a boy with chickenpox [1,2]. The VZV strain used for the vaccine generation is called Oka, consequently the parental and vaccine strains have been termed pOka and vOka, respectively. This live attenuated vOka vaccine was licensed for the prevention of chickenpox initially in the US in 1995 and since then in several mainly high income countries [93]. Since its introduction in the US, the vaccine has drastically reduced the number of varicella clinical cases and deaths. Moreover, the administration of a second dose of vaccine has significantly decreased breakthrough infections [94]. The vaccine is safe [95] and effective in preventing chickenpox or reducing the severity of it, as well as in building a long-last immunity against the virus [93]. However, being live, the vaccine is not suitable for administration to immunocompromised individuals [96]. The vOka vaccine, like wild-type VZV, stimulates the generation of antibodies as well as cell-mediated immunity (CMI). CMI is crucial for clearance of the virus once an individual has been infected, as the virus is highly cell-associated, as well as for the prevention of virus reactivation and HZ. However, CMI declines with age and this is believed to increase the risk of HZ in older age groups [94]. For this reason, the same vOka strain has been formulated at higher concentration for the prevention of HZ in older adults, as a therapeutic vaccine [97]. A newer glycoprotein E subunit vaccine adjuvanted with AS01_B_ adjuvant has recently been shown to be extremely effective in preventing HZ and its commonest sequela, post herpetic neuralgia [98].

Moffat et al. [7] demonstrated that vOka strain of VZV is attenuated for growth compared to pOka in the SCID-hu mouse skin xenograft model, although not in tissue culture monolayers [7,99]. No difference was found in the level of vOka titer recovered from infected T cells in the SCID-hu mouse model [3], indicating that the mechanism of vOka attenuation is likely to be reduced skin replication. Additionally, vOka appears to be impaired for reactivation [23,95]. Because the vOka strain has been produced by serial passaging of pOka in cells, it has accumulated a variety of genetic mutations, with 137 single-nucleotide polymorphisms (SNPs) shared by all the vOka vaccines and additional SNPs specific for certain commercial vaccines preparations, as identified by deep sequencing [100,101,102,103]. Only a few SNPs are at near fixation for the vaccine allele, with four present in the ORF62 gene and one in ORF0 [100,104,105]. ORF62 encodes the VZV transactivator IE62, which regulates the transcription of immediate early genes and is crucial in VZV skin pathogenesis [4]. ORF62 is the gene that in the vOka strain contains the major number of SNPs compared to pOka, of which four are highly fixed, with two of them causing R958G and S628G substitutions in two highly conserved regions of the ORF62 gene [106]. It also appears that vOka IE62 induces a decreased level of gene transcription compared to pOka IE62 [107]. All in all, the current available evidence suggests that IE62 may play an important role in the skin attenuation of the VZV vaccine and studies aimed at elucidating these mechanisms are underway.

Understanding the molecular mechanisms causing attenuation in skin of the existing VZV vaccine will shed light on pivotal events of VZV skin replication that could be targeted by new antiviral drugs, as well as pave the way for the generation of novel vaccines that could be directed to a wider number of recipients, including immunocompromised children.

## 6. Conclusions and Future Perspectives

Work on models that reproduce the architecture of human skin or at very least recapitulate keratinocyte differentiation has provided important insights into how VZV interacts with host epidermal pathways. In particular, the pivotal role of the specialised skin keratinocyte cell in the VZV replication cycle is likely to shed light on VZV tropism and pathogenesis. These mechanisms are just beginning to be unveiled and this work is also complicated by VZV infection being highly interconnected with the host epidermal biology, which itself is characterised by molecular pathways that have often been only partially elucidated. An example is epidermal autophagy, whose pathways have only quite recently begun to be uncovered [76,78]. An important tool to untangle such complexity and that could provide important insights into VZV infection at different stages of keratinocyte differentiation, is single cell RNA-sequencing, which has been already used successfully to identify cells at different stages of differentiation from human skin tissues [108,109]. This approach will allow to address the questions that remain unanswered concerning the role of epidermal differentiation in the VZV life cycle, particularly which differentiation pathways, together with K10 degradation, are implicated in VZV skin replication and blistering lesion formation. It will be important to elucidate the role of K15 and whether wound healing pathways are activated, at which stage of infection and for which purpose. Future studies will also need to address the molecular mechanisms and functions of autophagy and other cell stress processes in the context of VZV replication in the epidermis. Elucidation of these represents new druggable pathways for antiviral therapies and vaccine targets. For example, we have already shown that the small-molecule inhibitor nutlin-3, which is known to block MDM2 function [110], is able to restore K10 expression in VZV-infected keratinocytes and significantly reduce VZV growth in keratinocytes monolayers and skin explants [9]. A similar approach could be taken using existing inhibitors of autophagy.

Finally, a better understanding of VZV pathogenesis in the skin could help to develop new vaccines that are not based on the live attenuated virus. The existing vaccine vOka, being attenuated for growth in skin, is actually itself an important tool for the study of VZV skin infection and the comparative analysis with the parental strain pOka can further the understanding of the molecular mechanisms of VZV replication in skin. Moreover, a better knowledge of VZV skin pathogenesis will probably prove to be very valuable to further understand the pathogenesis of other skin epitheliotropic viruses that may share common mechanisms of replication in skin.
